# Consensus statements on complete mesocolic excision for right-sided colon cancer—technical steps and training implications

**DOI:** 10.1007/s00464-021-08395-0

**Published:** 2022-07-05

**Authors:** Patricia Tejedor, Nader Francis, David Jayne, Werner Hohenberger, Jim Khan, on behalf the CME Project Working Group, Patricia Tejedor, Nader Francis, David Jayne, Werner Hohenberger, Jim Khan

**Affiliations:** 1 Department of Colorectal Surgery, Queen Alexandra Hospital, Southwick Hill Road, Cosham, Portsmouth, PO6 3LY UK; 2 Department of Colorectal Surgery, Yeovil District Hospital, Yeovil, UK; 3 Division of Surgery and Interventional Science, University College London, London, UK; 4 Training Directorate At Griffin Institute Northwick Park Institute for Medical Research, London, UK; 5 Leeds Institute of Medical Research At St James’s, University of Leeds, Leeds, UK; 6 Department of Surgery, University Hospital Erlangen, Erlangen, Germany; 7 University of Portsmouth, Portsmouth, UK

**Keywords:** Complete mesocoloc excision, Right colon cancer, Expert consensus, Survival, Standardisation

## Abstract

**Background:**

CME is a radical resection for colon cancer, but the procedure is technically demanding with significant variation in its practice. A standardised approach to the optimal technique and training is, therefore, desirable to minimise technical hazards and facilitate safe dissemination. The aim is to develop an expert consensus on the optimal technique for Complete Mesocolic Excision (CME) for right-sided and transverse colon cancer to guide safe implementation and training pathways.

**Methods:**

Guidance was developed following a modified Delphi process to draw consensus from 55 international experts in CME and surgical education representing 18 countries. Domain topics were formulated and subdivided into questions pertinent to different aspects of CME practice. A three-round Delphi voting on 25 statements based on the specific questions and 70% agreement was considered as consensus.

**Results:**

Twenty-three recommendations for CME procedure were agreed on, describing the technique and optimal training pathway. CME is recommended as the standard of care resection for locally advanced colon cancer. The essential components are central vascular ligation, exposure of the superior mesenteric vein and excision of an intact mesocolon. Key anatomical landmarks to perform a safe CME dissection include identification of the ileocolic pedicle, superior mesenteric vein and root of the mesocolon. A proficiency-based multimodal training curriculum for CME was proposed including a formal proctorship programme.

**Conclusions:**

Consensus on standardisation of technique and training framework for complete mesocolic excision was agreed upon by a panel of experts to guide current practice and provide a quality control framework for future studies.

**Supplementary Information:**

The online version contains supplementary material available at 10.1007/s00464-021-08395-0.

Outcomes for patients with rectal cancer have improved following the acceptance of Total Mesorectal Excision (TME) surgery [1], whilst outcomes for patients with colonic cancer have remained fairly static. Right-sided colon cancers are associated with worse 5-year overall survival for stage II and III disease compared to rectal cancer [2]. This may be contributed to the variability of the technique of right hemicolectomy and the lack of standardisation of the oncology sound resection. Hohenberger was the first to publish a reduction in local recurrence and improved survival rates in patients with right-sided colon cancer undergoing complete mesocolic excision (CME) and central vascular ligation (CVL), based on the same TME concept and following the embryological planes but applied to the CME plane [3].

Whilst results of ongoing randomised control trials such as the ‘RELARC’ trial [4] and the Russian COLD trial [5] are still awaited, the evidence in Europe suggests a 15% increase in survival [6] and lower rates of 5-year local recurrence for stages I-III when this technique is adopted [7]. Additionally, retrospective studies have shown the importance of dissection in the correct mesocolic plane for the resection of colon cancer [8] as Heald et al. postulated for rectal cancer [1].

CME is a technically challenging surgical procedure with potential high morbidity [9–11] that requires intensive training. Lack of anatomical knowledge, gaps in surgical training, paucity of high-quality evidence and the potential morbidity has deterred surgeons from routinely adopting CME in their routine practice. Despite descriptions of institutionally standardised techniques and reports of its the practice from various surgeons, there is no international consensus on the standard technical requirements for CME [9], including standardising the terminology and anatomical landmarks and provide guidance on an agreed training platform for this demanding technique. Focussing on standardisation of surgical technique is shown to lead to higher rates of mesocolic resection and an oncologically superior specimen [12]. Developing consensus from an international expert panel on optimal training curriculum can also inform the development of training initiatives such as laparoscopic colorectal (Lapco) and transanal TME training programmes [13, 14].

The aim of this study was therefore, to establish an international expert consensus on a detailed structured description of the technique and the essential training framework for CME.

## Methods

A modified Delphi exercise was undertaken using a structured, reiterating questionnaire sent to a group of 55 international experts in Minimally Invasive Surgery (MIS), CME and surgical education representing 18 countries.

### Subject

The Steering Committee (SC) was formed consisting of PT, NF, DJ, WH and JK and tasked to coordinate the project. A CME Consensus Project Working Group (PWG) was assembled from 21 world experts in CME and education who attended an international collaborative CME workshop. The group reviewed the technique’s description and advised on the methodology and domains of this project. An additional 34 international experts were invited to join this project and contribute to the voting process. The steering group committee did not contribute to the voting process.

The selection of the expert panel was based on peer recommendation in the field of MIS in general and in CME procedure specifically. Their expertise spanned across a number of domains such as pioneers and early adopters of CME, experts in developing consensus and position statements and expertise in surgical education and developing training programmes. Details of the whole expert group are provided in Supplementary Appendix 1.

### CME workshop

This collaborative workshop took place at Portsmouth on 24-25th March 2019 bringing together international experts in CME to discuss the need and the main objectives of the project. The workshop involved 21 expert surgeons from with extensive experience in CME, as well as education leads who attended the workshop and formed the project working group.

During the workshop, which was chaired by JK and facilitated by the SC, the need for the project was discussed as well as the topic domains were proposed (nomenclature, operative steps, training and assessment). Questions were then formulated across the domains and twenty-five statements were drafted by the PWG addressing the questions across all domains. These were then finalised by the SC, to reduce redundancy and improve the readability but without influencing the meaning of each statement.

### Delphi process

Twenty-five statements were sent anonymously to the expert group using an electronic survey platform (www.surveymonkey.com) on 26th June 2019 (Supplementary Appendix 2). The results of the first round were analysed and sent out to each expert indicating the response from the first round. Items that reached consensus in the first round were removed and the remaining items were re-sent. Experts were informed of the consensus obtained in the previous round, so they knew the results before selecting a response in the next round. This iterative process aimed to achieve increasing consensus [15, 16]. To maintain anonymity of the process, experts were not aware of individual’s responses, only that of the whole group. This process was repeated for a maximum of 3 rounds until agreement was achieved. Agreement was defined as approval of the statements by 70% of the experts, which was considered a ‘majority positive verdict’, as proposed in the original Delphi description by Dalkey [16]. IRB approval was not required as this was an expert consensus.

## Results

The study was performed between May 2019 and May 2020. The response rate was 78% for the first round (43 responses), 69% for the second round and 69% for the third round (38 responses). The results of the different rounds are shown in Appendix 3.

Ultimately, the expert group agreed upon a total of 23 recommendations with a high-level agreement (over 70%), as shown in Supplementary Appendix 3. The recommendations were categorised according to defining the nomenclature, surgical technique, the training pathway and the assessment of performance. The level of agreement increased for all items, but 2 failed to reach consensus. The experts could not agree on a single best terminology to describe the operation but both CME or CME + CVL were proposed. The experts did, however, agreed that CVL was a key element of the procedure along with excision of an intact mesocolon. There was no agreement on the preferred approach for performing the technique.

## Nomenclature and surgical technique

Recommendation #1: The essential components of a procedure to qualify for CME are central vascular ligation (86% agreement), exposure of the superior mesenteric vein (SMV) (84% agreement) and excision of an intact mesocolon (92% agreement).

There was no agreement regarding the exposure of superior mesenteric artery (SMA) as a routine part of the CME procedure.

Recommendation #2: CME should be the standard of care resection for locally advanced colon cancer i.e., T3-4, N positive or circumferential margin threatened/affected (86% agreement).

Recommendation #3: CME is advisable for younger patients (under 50 years old) with a locally advanced colon cancer irrespective of the site (78% agreement).

Recommendation #4: Preoperative review of CT imaging and or reconstruction of vascular anatomy may be useful before undertaking CME, especially in minimally invasive surgery (92% agreement).

Recommendation #5: The key anatomical landmarks to perform a safe CME dissection include identification of the ileocolic pedicle, the SMV pedicle and root of the mesocolon (94% agreement).

Recommendation #6: In CME, the mesocolic fascia should be kept intact on both sides during colonic dissection (97% agreement).

Recommendation #7: CME surgery can be safely performed using either subileal, SMV first or supracolic approach, based on the surgeon’ preference (92% agreement).

Recommendation #8: A standard CME approach for caecal and ascending colon cancer may include omentectomy for technical rather than oncological reasons (78% agreement).

Recommendation #9: A standard CME approach for transverse and hepatic flexure colon cancer should include omentectomy (71% agreement).

Recommendation #10: For CME in transverse and hepatic flexure colon tumours, central ligation of the middle colic artery and vein at their origins from the superior mesenteric vessels is necessary (94% agreement).

Recommendation #11: In CME for right colon cancer, it is advisable to ligate the right colic vein (tributary of the Henle’s trunk) (97% agreement).

Recommendation #12: In CME for transverse colon cancer, including the flexures, it is advisable to ligate the right colic vein (tributary of the Henle’s trunk) (83% agreement).

There was no consensus regarding the management of the gastroepiploic vein in either right or transverse colon cancer.

Recommendation #13: In CME, routine central ligation of Henle’s trunk at its origin should be avoided (77% agreement).

Recommendation #14: Central ties should be marked with sutures/clips on the specimen (81% agreement).

### Training programme

Recommendation #15: A minimal experience of 50 laparoscopic conventional colon cancer resections is required prior to start CME training (90% agreement).

Recommendation #16: An expert/trainer in CME is defined by:

a workload and experience of performing CME (94% agreement)
b the provision of training courses, fellowship and proctoring in the field (100% agreement).

There was no agreement of the need for educational academic output as a requirement for CME trainers.

Recommendation #17: An optimal training curriculum for CME should include:

a Anatomy teaching (97% agreement),
b Case observation and video tutorial with an expert (94% agreement),
c Hands-on training course with a cadaver (83% agreement) and
d A formal proctorship programme (78% agreement).

### Assessment of performance

Recommendation #18: The optimal method to assess performance in CME should include:

a Specimen photographs (both sides-72% agreement),
b Video recording (74% agreement) and
c Review of pathological outcomes review (92% agreement).

Recommendation #19: Surgeons undertaking CME surgery should receive proficiency-based training assessed by:

a Clinical outcomes (76% agreement),
b Histological outcomes (82% agreement) and
c The review of video recorded cases using an objective assessment tools (86% agreement).

Recommendation #20: Surgeons undertaking CME training should demonstrate skill acquisition assessed by:

a Using global assessment score tools (78% agreement).
b Informal and structured feedback from the trainer (90% agreement),

Recommendation #21: Pictures of the resected central vessels area should be taken intraoperatively to ensure high-quality operation (86% agreement).

Recommendation #22: The surgeon/theatre team should measure the distance from the tumour to the high tie on the fresh specimen prior to fixation as a quality assurance of the specimen (78% agreement).

Recommendation #23: An international CME registry should be set up for data collection and audit (75% agreement).

## Discussion

The concept of CME, although not new, is gaining popularity amongst surgeons. Improved survival and reduced local recurrence rates have been reported in cohort studies and meta-analyses in favour of this technique compared to conventional right hemicolectomy [3, 7]. The barriers to the wider adoption of CME have included concerns about the technical difficulties of the operation and the likelihood of a higher complication rate, such as delayed gastric emptying [11]. Variations in terminology and techniques make it difficult to compare the published literature and to design a clinical research study or training curriculum as there has to be a clear and standardised surgical approach to CME. This international consensus project was undertaken to bring together expert opinion and agree on the critical steps of the technique and the essential elements of a training pathway to support safe adoption and wider implementation of CME.

This project achieved its aims in clarifying the terminology related to the specific components of CME and identifying the indications for CME for locally advanced colon cancer, especially among younger patients. This indication can be justified by the potential oncological benefits for younger patients, using an advanced technique. Nevertheless, indication for this procedure should be made on an individual basis and within the context of overall patient fitness and comorbidities rather than just the chronological age. Practically its very hard for a surgeon to offer a CME and a non-CME procedure for similar colonic cancer patients and when a unit embraces CME technique in their practice, its becomes the standard of care for all comers. Similarly the radiological staging of locally advanced cancers is not without limitations. Nodal staging is associated with a significantly false positive rate and there is a clear need for better understanding of radiological criteria for diagnosis of such cases.

The technical steps of CME have been described by previous studies [3, 17]. Our project is novel in that it uses a robust methodology with adherence to Delphi process of establishing expert consensus to achieve consensus from a broad range of international experts with a wide variation of expertise including training and education. A high level of agreement on the technical steps was reached by the experts and is presented as recommendation. Nearly 100% of the expert group agreed that the key anatomical landmarks for a safe CME should include identification of the ileocolic pedicle, SMV pedicle and root of mesocolon; as previously reported by Hohenberger [3]. Routine central ligation of Henle’s trunk at its origin should be avoided and it is advisable to ligate the right colic vein. Contrary to the original recommendation for CME for transverse colon tumours, [3] central ligation of the gastroepiploic vein in either right or transverse colon cancer was not agreed on. Alternatively, the experts advised to ligate the right colic vein (tributary of the Henle’s trunk).

There is little evidence to support a particular surgical approach for CME such as open, laparoscopic or robotic [18]; the experts agreed that CME is feasible by all three approaches and the choice should be left to the surgeon’s preference based on training and local resources.

Standardisation of this complex technique is essential not only to provide quality assurance of technique that aims to reduce major complications, but to inform training platforms. The experts highlighted the real need for a structured training pathway that encompasses assessment of performance to promote quality standards, as described in the summary of recommendations.

It is difficult to establish a minimum requirement for the learning curve of CME. Guo et al. demonstrated CME proficiency could be attained after 25 cases [19]. The recommendation of having experience of at least 50 colonic resection prior to undertaking CME surgery is to ensure that the surgeons have sufficient experience before undertaking a more complex procedure. However, in units where CME is the standard of care for right colon cancer, trainee may not be exposed to non-CME surgery at all. A pragmatic approach will have to be undertaken in these scenarios to offer a safe learning experience. A multimodal training curricula has been proposed by the experts to enhanced learning. An optimal training curriculum for CME should include appraisal of the anatomy of the right colon, including the vascular supply. Case observation with video tutorial by expert was also recommended in addition to hands-on training courses with cadaver models. A structured proctorship programme was highly recommended as it has been found to be essential in other surgical training programmes [13, 14] to improve the efficiency and efficacy of learning.

In order to assess performance in CME, the experts group recommended taking specimen photographs immediately after the operation to evaluate the length of both the anterior and posterior mesocolic surfaces, along with a metric scale for calibration and measurement of the distance from the tumour to the high vascular tie [20]. The experts agreed that these measurements were important, but there was no agreement on the need for recording the length of resected colon, the mesenteric area or the distance from the bowel to the high vascular tie, despite the previous literature recommending a colonic resection 10 cm beyond the tumour [21, 22]. Additionally, intraoperative photographs of the dissected field were recommended by the experts. These images should capture the area of central vessels division to ensure complete CME had been performed. The expert group encouraged video recording of all procedures and to be used as a platform to provide a structured feedback assessment to the learners. Review of pathological outcomes was agreed on by the vast majority of our expert panel as the optimal method to assess performance in CME. An international registry for data collection was highly recommended to audit outcomes and serve as a resource for further studies.

A limitation of this study is the reliance on expert opinion, inherent in the Delphi process, to inform recommendations rather than primary research. A robust consensus seeking process was followed in this project to overcome the inherent limitations to group pooling and discussion by virtue of its structure and element of anonymity. This facilitated controlled feedback, reiterated of concept and reassessed opinions until a final consensus was achieved according to the original description of Delphi technique [16]. Nevertheless the agreed recommendations represent the opinion of this group of experts and further research is required to assess their impact. Experts’ selection is another critical issue with consensus statements. The selection of our experts was based on peer recommendation of recognised expertise in the field with a wide distribution of expertise in education, development of consensus and position statements as well as training initiatives from across Europe and beyond to ensure the broadest scope of opinion. In this study, the response rate among the experts was relatively high (over 70%) in each round compared to other similar studies, indicating their commitment to reach consensus. Additionally, the contribution of the steering and consensus development groups of data collection could arguably dilute or alter the original intent. Although theses panels helped to reduce redundancy by combining and clarifying the questions, which are requirements of the Delphi process, the review panel made every effort in preserving the initial questions’ intent.

CME for right-sided colon cancer is becoming more popular worldwide, making this consensus statement necessary and timely. With further adoption of the technique, international collaborative efforts are required to develop and implement training initiative in CME that are underpinned by the agreed training recommendations. The proposed statements should also provide a quality control framework for future research that can fully appraise the impact of this technique as well as the effectiveness of training programmes of CME.

## Conclusions

A consensus on Complete Mesocolic Excision has been achieved by a panel of experts to guide current practice and promote optimal training. These recommendations have the potential to provide quality control framework for future CME research initiatives.

## Supplementary Information

Below is the link to the electronic supplementary material.

Supplementary file1 (DOCX 158 KB)

Supplementary file2 (DOCX 110 KB)

Supplementary file3 (DOCX 32 KB)
