# Supplementary material for: Consensus statements on complete mesocolic excision for right-sided colon cancer—technical steps and training implications
Source: Surg Endosc. 2022 Jul 5;36(8):5595–601. doi: 10.1007/s00464-021-08395-0 (PMC9283340; doi:10.1007/s00464-021-08395-0)
Supplement: Supplementary file 1 — Supplementary file1 (DOCX 158 KB) [file 464_2021_8395_MOESM1_ESM.docx]

Appendix 1. Details of the CME expert participating group:

1. CME Consensus Steering Group (SG):
2. Patricia Tejedor. Department of colorectal surgery, Queen Alexandra Hospital, Portsmouth, UK.
3. Nader Francis. Department of colorectal surgery, Yeovil District Hospital, UK / Division of Surgery and Interventional Science, University College London, UK / Training Directorate at Griffin Institute Northwick Park Institute for Medical Research, London, UK.
4. David Jayne. Leeds Institute of Medical Research at St James's, University of Leeds, Leeds, UK.
5. Werner Hohenberger. Department of Surgery, University Hospital Erlangen, Erlangen, Germany.
6. Jim Khan. Department of colorectal surgery, Queen Alexandra Hospital, Portsmouth, UK / Anglia Ruskin University Chelmsford, UK.
7. CME Consensus Project Working Group (PWG):
   1. Anwar Ahmed, Basildon Hospital, UK
   2. Chukwuma Abraham-igwe, Department of Colorectal Surgery, University Hospitals Birmingham- HGS, West Midlands, UK
   3. Charles Evans, University Hospitals Coventry, UK
   4. Danilo Miskovic. St. Mark’s Hospital, London, UK
   5. Frank Pfeffer. Department of Gastrointestinal and Emergency Surgery, Haukeland University Hospital, Bergen, Norway
   6. Inna Tulina, Clinic of Colorectal and Minimally Invasive Surgery, Sechenov First Moscow State Medical University
   7. John Calvin Coffey, Department of Surgery, University Hospital Group Limerick, Ireland 4i Centre for interventions in infection, inflammation and immunity, School of Medicine, University of Limerick, Ireland
   8. Kenneth Campbell, Nine-wells Hospital Dundee, UK
   9. Marcos Gómez-Ruiz. Colorectal Unit, University Hospital Marqués de Valdecilla, Santander, Spain / Institutio de Investigación Biomédica IDIVAL
   10. Nicholas Paul West. Pathology & Data Analytics, Leeds Institute of Medical Research at St. James’s, University of Leeds, Leeds, UK
   11. Oliver Shihab, Portsmouth Hospitals NHS trust UK
   12. Paolo Pietro Bianchi, Department of Surgery Ospedale Misericordia Grosseto Italy
   13. [Peter Edward Coyne.](mailto:peter.coyne@nuth.nhs.uk)  Royal Victoria Infirmary, Newcastle upon Tyne Hospitals NHS Foundation Trust. UK
   14. Petr Tsarkov, Colorectal & Minimal Invasive Surgery, Sechenov University, Moscow, Russia
   15. Philip Varghese, Department of Colorectal Surgery University Hospitals of North Midlands NHS Trust, Stoke-on-Trent. UK
   16. Roger W. Motson, Professor of Surgery, The ICENI Centre, Anglia Ruskin University
   17. Roger Gerjy, Consultant Colorectal and General Surgeon Mediclinic City Hospital Dubai, UAE
   18. Samson Tou. University Hospitals of Derby and Burton NHS Foundation Trust
   19. Talvinder Singh Gill, Department of Surgery, University Hospital of North Tees, Stockton on Tees, UK
   20. Tsuyoshi Konishi, Department of Gastroenterological Surgery, Cancer Institute Hospital of the Japanese Foundation for Cancer Research
   21. Volkan Ozben. Department of General Surgery, Acibadem Mehmet Ali Aydinlar University, School of Medicine, Istanbul, Turkey
8. CME Consensus Expert Group:
9. Alberto Arezzo, Department of Surgical Sciences, University of Torino, Italy
10. Andrew RL Stevenson. Royal Brisbane Hospital, University of Queensland , Brisbane, Australia
11. Andrew Craig Lynch, Department of Surgery, St Vincent’s Hospital, University of Melbourne, Melbourne, Australia
12. Byung Soh Min. Department of Surgery, Yonsei University College of Medicine, Seoul, Korea
13. Carlos Pastor. University Clinic of Navarre, Madrid, Spain.
14. Claus Anders Bertelsen, Department of Surgery, Nordsjællands Hospital, Dyrehavevej 29, 3400 Hillerød, Denmark Department of Clinical Medicine, Faculty of Health and Medical Sciences, Copenhagen University, 2200 Copenhagen, Denmark
15. Dieter Hahnloser, Department of Visceral Surgery, University Hospital Lausanne, Switzerland
16. Dejan Ignjatovic, Department of Digestive Surgery, Akershus University Hospital, University of Oslo, Lorenskog, Norway
17. Giuseppe Spinoglio. Candiolo Cancer Institute-FPO, IRCCS, Candiolo (Torino), Italy
18. Hermann Kessler, Department of Colorectal Surgery, Digestive Disease and Surgery Institute, Cleveland Clinic
19. Hirotoshi Hasegawa, MD FRCS FASCRS, Department of Surgery, Tokyo Dental College Ichikawa General Hospital Ichikawa City Japan
20. Koji Okabayashi, Department of Surgery, Keio University School of Medicine, Tokyo, Japan
21. Luigi Boni. Fondazione IRCCS - Ca' Granda - Ospedale Maggiore Policlinico di Milano, University of Milan, Italy.
22. [Mario Morino.](mailto:mario.morino@unito.it)  Department of Surgery, University of Torino, Italy
23. Michael R. Leitz, Surgical Oncology Unit, Department of General and Visceral Surgery, Klinikum Lippe, Germany
24. Rogier MPH Crolla, MD, Amphia Hospital Breda, the Netherlands.
25. Roland S. Croner, Department of General-, Visceral-, Vascular- and Transplant Surgery, University Hospital Magdeburg, Germany
26. Seon Hahn Kim, Anam University Hospital, Korea
27. Sergey K. Efetov, Clinic of colorectal and minimally invasive surgery, I M Sechenov First Moscow State Medical University, Moscow, Russia
28. Stefan R. Benz. Department of General-, Visceral-, and Pediatric Surgery, Klinikum Böblingen-Sindelfingen, Germany
29. Stefan Niebisch, University of Leipzig Medical Center, Department of Surgery, Leipzig, Germany
30. [Yves Panis.](mailto:yves.panis@gmail.com)  Beaujon Hospital, Clichy, and University of Paris, France
31. Wolfgang F.A. Hiller, Klinik für Allgemein- und Visceralchirurgie Klinikum Lippe gmbh Röntgenstr. Germany
32. Willem Bemelman. Academic Medical Centre, University of Amsterdam, Amsterdam, Netherlands.
