# Supplementary material for: Consensus statements on complete mesocolic excision for right-sided colon cancer—technical steps and training implications
Source: Surg Endosc. 2022 Jul 5;36(8):5595–601. doi: 10.1007/s00464-021-08395-0 (PMC9283340; doi:10.1007/s00464-021-08395-0)
Supplement: Supplementary file 2 — Supplementary file2 (DOCX 110 KB) [file 464_2021_8395_MOESM2_ESM.docx]

Appendix 2. 25-item questionnaire developed and sent to the experts group.

SMV: Superior Mesenteric Vein. SMA: Superior Mesenteric Artery. CME: Complete Mesocolic Excision. CRM: Circumferential Resection Margin. MIS: Minimally Invasive Surgery. GCT: Gastrocolic Trunk

| 1. What is the best terminology to describe the operation which involves dissection along the anterior surface of SMV and excision of lymphatic tissue and central ligation of vessels. |
| --- |
| - Complete mesocolic excision |
| - Complete mesocolic excision + Central Vascular Ligation |
| - D3 lymphadenectomy |
| 1. What are the essential components of the procedure to qualify for CME? |
| - Include central vascular ligation |
| - Exposure of SMV |
| - Exposure of SMA (for surgical orientation) |
| - Excision of intact mesocolon |
| - Dissection around SMA |
| 1. CME should be the standard of care resection for locally advanced colon cancer (T3-4, N1-N2, CRM+)  - Yes - No |
| 1. CME is advisable for a younger cohort of patients (under 50 ys) with a locally advanced colon cancer, irrespective of the site.  - Yes - No |
| 1. Preoperative review of CT imaging and or reconstruction of vascular anatomy may be useful before undertaking CME surgery, especially in MIS approach.  - Yes - No |
| 1. What is the preferred approach for CME surgery in your practice? |
| - Open |
| - Laparoscopic |
| - Robotic |
| 1. The key anatomical landmarks to start a safe CME dissection include identification of ileocolic pedicle, SMV pedicle, and root of mesocolon.  - Yes - No |
| 1. In CME, the mesocolic fascia should be kept intact on both sides after colonic resection.  - Yes - No |
| 1. CME surgery can be safely performed using subileal, SMV first or supracolic approach based on surgeons’ preference.  - Yes - No |
| 1. A standard CME approach for caecal and ascending colon cancer may include omentectomy for technical rather than oncological reasons.  - Yes - No |
| 1. A standard CME approach for transverse and haepatic flexure colon cancer should include omentectomy.  - Yes - No |
| 1. For CME in transverse & flexures colon tumours, central ligation of the middle colic artery and vein at their origins from superior mesenteric vessels is necessary.  - Yes - No |
| 1. In CME for right colon cancer, its advisable to ligate the following tributaries of the Henle’s trunk (GCT) |
| - Right colic vein |
| - Gastroepiploic vein |
| 1. In CME for transverse colon cancer, including the flexures, its advisable to ligate the following tributaries of the Henle’s trunk (GCT) |
| - Right colic vein |
| - Gastroepiploic vein |
| 1. In CME, routine central ligation of the Henle’s trunk at its origin should be avoided.  - Yes - No - Don’t know |
| 1. Central ties should be marked with sutures/clips on the specimen.  - Yes - No |
| 1. Defining the learner; what is the minimal experience of laparoscopic colon cancer surgery that is required prior to CME training? |
| - > 20 |
| - > 50 |
| - > 100 |
| 1. Defining the trainer; the following criteria defines CME trainers/ experts |
| - Workload and experience in CME |
| - Provision of training courses/fellowship/proctoring in the field |
| - Educational academic output in the field |
| 1. Implementation of training; the following criteria defines an optimal training curriculum for CME |
| - Teaching anatomy |
| - Case observation and video tutorial with the expert |
| - Hands on training course using simulation (cadaver) |
| - Formal proctorship program |
| 1. What is the optimal method to assess performance in CME? |
| - Specimen photographs (both sides) should be taken for quality control |
| - Clinical outcomes |
| - Pathological outcomes |
| - Video recording |
| 1. Surgeon undertaking CME surgery should receive proficiency based training assessed by: |
| - Clinical outcomes including morbidity and mortality |
| - Histological outcomes |
| - Review of video recorded cases using objective assessment tools |
| 1. Surgeon undertaking CME training should demonstrate knowledge and skill acquisition assessed by: |
| - Informal feedback from trainer during or after surgery |
| - Structured feedback from trainer using work based assessment tools or global assessment score tools |
| - Passing knowledge based module that is specific for this surgery (which will need to be developed) |
| 1. An international CME registry should be set up for data collection and audit.  - Yes - No |
| 1. The following measurements should be recorded with fresh specimen prior to fixation (by local surgeon/theatre team with metric scale present): |
| - Distance tumour to high tie |
| - Distance bowel to high tie |
| - Length of colon removed |
| - Mesenteric area |
| 1. Intraoperatively, pictures of the resected central vessels area should be taken to ensure high quality operation.  - Yes - No |
