# Supplementary material for: Consensus statements on complete mesocolic excision for right-sided colon cancer—technical steps and training implications
Source: Surg Endosc. 2022 Jul 5;36(8):5595–601. doi: 10.1007/s00464-021-08395-0 (PMC9283340; doi:10.1007/s00464-021-08395-0)
Supplement: Supplementary file 3 — Supplementary file3 (DOCX 32 KB) [file 464_2021_8395_MOESM3_ESM.docx]

Appendix 3. Results of the three rounds of the online survey.

SMV: Superior Mesenteric Vein. SMA: Superior Mesenteric Artery. CME: Complete Mesocolic Excision. CRM: Circumferential Resection Margin. MIS: Minimally Invasive Surgery. GCT: Gastrocolic Trunk

| Item | Round 1 | Round 2 | Round 3 |
| --- | --- | --- | --- |
| **Nomenclature and surgical technique** | | | |
| 1. What is the best terminology to describe the operation which involves dissection along the anterior surface of SMV and excision of lymphatic tissue and central ligation of vessels. | | | |
| Complete mesocolic excision | 39.5% | 50% | 55.2% |
| Complete mesocolic excision + Central Vascular Ligation | 46.5% | 39.5% | 42.1% |
| D3 lymphadenectomy | 18.6% | 10.5% | 5.2% |
| 1. What are the essential components of the procedure to qualify for CME? | | | |
| Include central vascular ligation | 88.3% | - | - |
| Exposure of SMV | 69.7% | 84.2% | - |
| Exposure of SMA (for surgical orientation) | 23.2% | 15.8% | 42.1% |
| Excision of intact mesocolon | 90.7% | - | - |
| Dissection around SMA | 6.9% | 7.9% | 7.9% |
| 1. CME should be the standard of care resection for locally advanced colon cancer (T3-4, N1-N2, CRM+) | 88.3% | - |  |
| 1. CME is advisable for a younger cohort of patients (under 50 ys) with a locally advanced colon cancer, irrespective of the site. | 81.4% | - |  |
| 1. Preoperative review of CT imaging and or reconstruction of vascular anatomy may be useful before undertaking CME surgery, especially in MIS approach. | 93% | - |  |
| 1. What is the preferred approach for CME surgery in your practice? | | | |
| Open | 16.7% | 16% | 15.8% |
| Laparoscopic | 52.4% | 49.6% | 50% |
| Robotic | 28.5% | 27% | 42% |
| 1. The key anatomical landmarks to start a safe CME dissection include identification of ileocolic pedicle, SMV pedicle, and root of mesocolon | 92.8% | - | - |
| 1. In CME, the mesocolic fascia should be kept intact on both sides after colonic resection. | 97.7% | - | - |
| 1. CME surgery can be safely performed using subileal, SMV first or supracolic approach based on surgeons’ preference | 90.7% | - | - |
| 1. A standard CME approach for caecal and ascending colon cancer may include omentectomy for technical rather than oncological reasons. | 81.4% | - | - |
| 1. A standard CME approach for transverse and haepatic flexure colon cancer should include omentectomy | 72% | - | - |
| 1. For CME in transverse & flexures colon tumours, central ligation of the middle colic artery and vein at their origins from superior mesenteric vessels is necessary | 95.3% | - | - |
| 1. In CME for right colon cancer, its advisable to ligate the following tributaries of the Henle’s trunk (GCT) | | | |
| Right colic vein | 95.3% | - | - |
| Gastroepiploic vein | 11.9% | 4% | 2.6% |
| 1. In CME for transverse colon cancer, including the flexures, its advisable to ligate the following tributaries of the Henle’s trunk (GCT) | | | |
| Right colic vein | 85.7% | - | - |
| Gastroepiploic vein | 52.4% | 36.8% | 34.2% |
| 1. In CME, routine central ligation of the Henle’s trunk at its origin should be avoided. | 77.8% | - | - |
| 1. Central ties should be marked with sutures/clips on the specimen | 81.4% | - | - |
| **Training pathway** | | | |
| 1. Defining the learner; what is the minimal experience of laparoscopic colon cancer surgery that is required prior to CME training? | | | |
| > 20 | 32.6% | 26.3% | 7.9% |
| > 50 | 39.5% | 68.4% | 89.5% |
| > 100 | 20.9% | 5.2% | 0% |
| 1. Defining the trainer; the following criteria defines CME trainers/ experts | | | |
| Workload and experience in CME | 93% | - | - |
| Provision of training courses/fellowship/proctoring in the field | 62.8% | 100% | - |
| Educational academic output in the field | 32.6% | 13.5% | 28.9% |
| 1. Implementation of training; the following criteria defines an optimal training curriculum for CME | | | |
| Teaching anatomy | 95.3% | - | - |
| Case observation and video tutorial with the expert | 90.7% | - | - |
| Hands on training course using simulation (cadaver) | 83.7% | - | - |
| Formal proctorship program | 79.1% | - | - |
| **Assessment of performance** | | | |
| 1. What is the optimal method to assess performance in CME? | | | |
| Specimen photographs (both sides) should be taken for quality control | 76.7% | - | - |
| Clinical outcomes | 39.5% | 18.4% | 26.3% |
| Pathological outcomes | 62.8% | 65.7% | 92.1% |
| Video recording | 67.4% | 73.6% | - |
| 1. Surgeon undertaking CME surgery should receive proficiency based training assessed by: | | | |
| Clinical outcomes including morbidity and mortality | 65.1% | 76.3% | - |
| Histological outcomes | 67.4% | 81.6% | - |
| Review of video recorded cases using objective assessment tools | 88.4% | - | - |
| 1. Surgeon undertaking CME training should demonstrate knowledge and skill acquisition assessed by: | | | |
| Informal feedback from trainer during or after surgery | 46.5% | 89.5% | - |
| Structured feedback from trainer using work based assessment tools or global assessment score tools | 79.1% | - | - |
| Passing knowledge based module that is specific for this surgery (which will need to be developed) | 34.9% | 36.8% | 55.2% |
| 1. An international CME registry should be set up for data collection and audit. | 76.7% | - | - |
| 1. The following measurements should be recorded with fresh specimen prior to fixation (by local surgeon/theatre team with metric scale present): | | | |
| Distance tumour to high tie | 76.7% | - |  |
| Distance bowel to high tie | 65.1% | 61.3% | 52.6% |
| Length of colon removed | 60.5% | 60% | 42.1% |
| Mesenteric area | 55.8% | 54.2% | 52.6% |
| 1. Intraoperatively, pictures of the resected central vessels area should be taken to ensure high quality operation. | 88.4% | - |  |
